# *Yinanyphaena* gen. nov., a new ghost spider genus with descriptions of five new species and seven new combinations from China (Araneae, Anyphaenidae)

**DOI:** 10.3897/zookeys.1294.203456

**Published:** 2026-10-09

**Authors:** Jincan Zhang, Zegang Feng, Jiahui Gan, Cheng Wang, Xiaoqi Mi

**Affiliations:** 1 Guizhou Provincial Key Laboratory for Biodiversity Conservation and Utilization in the Fanjing Mountain Region, Tongren University, Tongren, Guizhou 554300, China Guizhou Provincial Key Laboratory for Biodiversity Conservation and Utilization in the Fanjing Mountain Region, Tongren University Guizhou China; 2 Central South Academy of Inventory and Planning of National Forestry and Grass Administration, Changsha 410014, Hunan, China Central South Academy of Inventory and Planning of National Forestry and Grass Administration Changsha China; 3 School of Life and Health Science, Kaili University, Kaili 556011, Guizhou, China School of Life and Health Science, Kaili University Guizhou China

**Keywords:** Diversity, morphology, multigene, new combination, new genus, new species, taxonomy, South China

## Abstract

A new genus, *Yinanyphaena* Wang & Mi, **gen. nov**., of the family Anyphaenidae Bertkau, 1878, is proposed based on molecular phylogenetic results and morphological evidence. Five new species collected from the Qomolangma National Nature Reserve, Xizang, China are described: *Y. haochuni* Wang & Mi, **sp. nov**. (♂♀), *Y. jinhuai* Wang & Mi, **sp. nov**. (♂♀), *Y. qomolangma* Wang & Mi, **sp. nov**. (♂♀), *Y. shikaii* Wang & Mi, **sp. nov**. (♀), and *Y. xufeii* Wang & Mi, **sp. nov**. (♀). In addition, seven new combinations transferred from *Anyphaena* Sundevall, 1833 are also proposed: *Y. cibagou* (Wang & Mi, 2024), **comb. nov**., *Y. linzhi* (Wang & Mi, 2024), **comb. nov**., *Y. rhynchophysa* (Feng, Ma & Yang, 2012), **comb. nov**., *Y. shufui* (Wang & Mi, 2024), **comb. nov**., *Y. tibet* (Lin & Li, 2021), **comb. nov**., *Y. wuyi* (Zhang, Zhu & Song, 2005), **comb. nov**., and *Y. yejiei* (Wang & Mi, 2024), **comb. nov**. Diagnostic photos of the new species and *Y. wuyi*, along with a distributional map for the genus are provided.

## Introduction

The family Anyphaenidae Bertkau, 1878, commonly known as ghost spiders, comprises a diverse group of nocturnal, cursorial spiders that actively hunt on vegetation (Labarque et al. 2015). 663 valid species across 59 genera have been recorded, most of which are found in the Americas (WSC 2026). In China, the diversity of this family is low, with only 16 species from three genera documented to date, though this is still much higher than in nearby countries (Li et al. 2024; WSC 2026). Of these, four species are placed in two new genera erected by Lin et al. (2021, 2022), while the remaining species belong to *Anyphaena* Sundevall, 1833, the most diverse genus within the family.

Among known Chinese species of *Anyphaena*, most exhibit copulatory organs that differ considerably from those of the type species, particularly the species recently reported from Xizang. During our recent surveys in the Qomolangma National Nature Reserve in Xizang and the Fanjing Mountain National Nature Reserve in Guizhou, several congeners of those species were also collected. To further clarify their generic placement, we conducted a molecular phylogenetic analysis, and the results support that they are not members of *Anyphaena*. Additional morphological comparisons indicate that they do not belong to any currently recognized genus. On this basis, we propose a new genus, *Yinanyphaena* Wang & Mi, gen. nov., including five new species described herein and seven new combinations transferred from *Anyphaena*.

## Materials and methods

Specimens were collected by beating shrubs. They were preserved in 95% ethanol and deposited at Tongren University (**TRU**), Tongren, China.

### Molecular data

The legs of the specimens of selected species were used to extract genomic DNA and sequence four gene fragments: COI, 18S, 28S, and H3. Primer pairs and PCR protocols are given in Table 1. DNA extraction methods follow Wang et al. (2025). PCR amplification included a 2 min 95 °C initial denaturation and 35 iterations of 30 s at 95 °C, 30 s annealing step at 48 °C (COI), 50 °C (18S), 56 °C (H3) and 62 °C (28S), 30 s at 72 °C and one 10 min extension step at 72 °C. Sequencing of the PCR products was done at Tsingke (Changsha, China).

**Table 1.** Primers and amplification conditions used in the PCR.

**Locus**	**Annealing temperature/time**	**Direction**	**Primer**	**Sequence 5'–3'**	**References**
18S	50°/30s	F	1F	TACCTGGTTGATCCTGCCAGTAG	Giribet and Ribera 2000
		R	5R	CTTGGCAAATGCTTTCGC	Giribet et al. 1996
28S	62°/30s	F	28SO	GAAACTGCTCAAAGGTAAACGG	Whiting et al. 1997
		R	28SC	GGTTCGATTAGTCTTTCGCC	
COI	48°/30s	F	LCO1490	GGTCAACAAATCATAAAGATATTGG	Folmer et al. 1994
		R	HCO2198	TAAACTTCAGGGTGACCAAAAAATCA	
H3	56°/30s	F	H3aF	ATGGCTCGTACCAAGCAGACV	Colgan et al. 1998
		R	H3aR	ATATCCTTRGGCATRATRGTG	

A total of 33 individuals belonging to 26 species were used for the phylogenetic analysis (Table 2). The ingroup includes 20 known species and five new species of *Yinanyphaena* gen. nov. *Malenella nana* Ramírez, 1995 was used as the outgroup. This species has recently been transferred to Macrobunidae Bonnet, 1957 (Gorneau et al. 2023), but this reclassification does not affect the ingroup topology presented.

**Table 2.** Species and their DNA data for molecular phylogenetic analysis.

**Species**	**Molecular voucher**	**COI**	**18S**	**28S**	**H3**
*Acanthoceto acupicta*	F31/MACN‐Ar 12068	MZ902411		KR558884	KR558767
*Acanthoceto pichi*	E67/MACN‐Ar 28390	MZ902396		KR558887	KR558770
*Anyphaena accentuata*	ARASP000039	KY268940	KY016299	KR558945	KR558829
*Anyphaenoides clavipes*	F19	KR558955		KR558889	KR558772
*Aysenia elongata*	F09/MACN‐Ar 10869	KR558961		KR558895	KR558778
*Malenella nana*	ARAMR000257/MACN‐Ar 28842	KR558977		KR558910	KR558794
*Negayan excepta*	-	MZ902489			KX817551
*Negayan paduana*	ARAMR000062	MZ902455	KY016307	KR558946	KR558830
*Otoniela adisi*	F01/MACN‐Ar 10981	KR558984		KR558947	
*Philisca accentifera*	F32/MACN‐Ar 10867	KR558987		KR558919	KR558803
*Philisca doilu*	F14/MACN‐Ar 10934	KR558989		KR558921	KR558805
*Philisca tripunctata*	F05/MACN‐Ar 28846	KR558996		KR558928	KR558812
*Tomopisthes horrendus*	F94/MACN‐Ar 30583	KR559009		KR558942	KR558826
*Tomopisthes varius*	ARAMR000059	KR559014	KY016313	KR558948	KR558831
*Yinanyphaena cibagou*	TRU-XZ-ANY-0001	PP356956			
*Yinanyphaena linzhi*	TRU-XZ-ANY-0005	PP356958			
*Yinanyphaena shufui*	TRU-XZ-ANY-0014	PP356960			
*Yinanyphaena yejiei*	TRU-XZ-ANY-0017	PP356959			
*Anyphaena leidashi*	A31	**PZ869189**			**PZ872375**
*Anyphaena leidashi*	A36	**PZ869190**			**PZ872376**
*Yinanyphaena haochuni*	A13	**PZ869180**	**PZ869462**	**PZ869472**	**PZ872369**
*Yinanyphaena haochuni*	A14	**PZ869181**	**PZ869463**	**PZ869473**	**PZ872370**
*Yinanyphaena jinhuai*	A19	**PZ869183**			
*Yinanyphaena jinhuai*	A20	**PZ869184**			
*Yinanyphaena linzhi*	A38	**PZ869191**	**PZ869468**	**PZ869476**	**PZ872377**
*Yinanyphaena qomolangma*	A29	**PZ869187**	**PZ869466**		**PZ872373**
*Yinanyphaena qomolangma*	A30	**PZ869188**	**PZ869467**		**PZ872374**
*Yinanyphaena shikaii*	A27	**PZ869186**		**PZ869475**	**PZ872372**
*Yinanyphaena tibet*	A18	**PZ869182**	**PZ869464**	**PZ869474**	
*Yinanyphaena tibet*	A55	**PZ869194**	**PZ869471**	**PZ869477**	
*Yinanyphaena wuyi*	A41	**PZ869192**	**PZ869469**		**PZ872378**
*Yinanyphaena wuyi*	A44	**PZ869193**	**PZ869470**		**PZ872379**
*Yinanyphaena xufeii*	A25	**PZ869185**	**PZ869465**		**PZ872371**

Sequences were aligned with MAFFT (Katoh and Standley 2013) and were concatenated in PhyloSuite (Zhang et al. 2020). ModelFinder (Kalyaanamoorthy et al. 2017) was used to identify the best-fit molecular evolution models for each locus as follows: COI, 28S, H3: SYM+I+G4; 18S: JC+I.

The maximum likelihood (ML) tree was constructed using IQ-TREE 2.0 (Minh et al. 2020) with default parameters and 5000 bootstrap replicates, and was finally edited and processed in the iTOL (https://itol.embl.de/).

### Morphological data

Methods of specimen examination, photographs of specimens stored in alcohol and distributional map generation follow Li et al. (2024). Photographs of living spiders were taken with a Canon EOS R7 camera equipped with a Canon RF100 mm F2.8 L MACRO IS USM lens. Leg measurements are given as: total length (femur, patella, tibia, metatarsus, tarsus). Descriptions below are based on the specimens deposited in alcohol. Abbreviations used in the text and figures are as follows: **AG** accessory gland; **ALE** anterior lateral eye; **AME** anterior median eye; **At** atrium; **CD** copulatory duct; **CO** copulatory opening; **dRTA** dorsal ramus of retrolateral tibial apophysis; **E** embolus; **FD** fertilization duct; **MA** median apophysis; **MOA** median ocular area; **PLE** posterior lateral eye; **PME** posterior median eye; **PPA** prolateral patellar apophysis; **PTA** prolateral tibial apophysis; **vRTA** ventral ramus of retrolateral tibial apophysis; **VTA** ventral tibial apophysis; **VTP** ventral tegular process; **S** spermatheca; **St** subtegulum; **T** tegulum.

## Results

### Phylogenetic results

**Figure 1.**
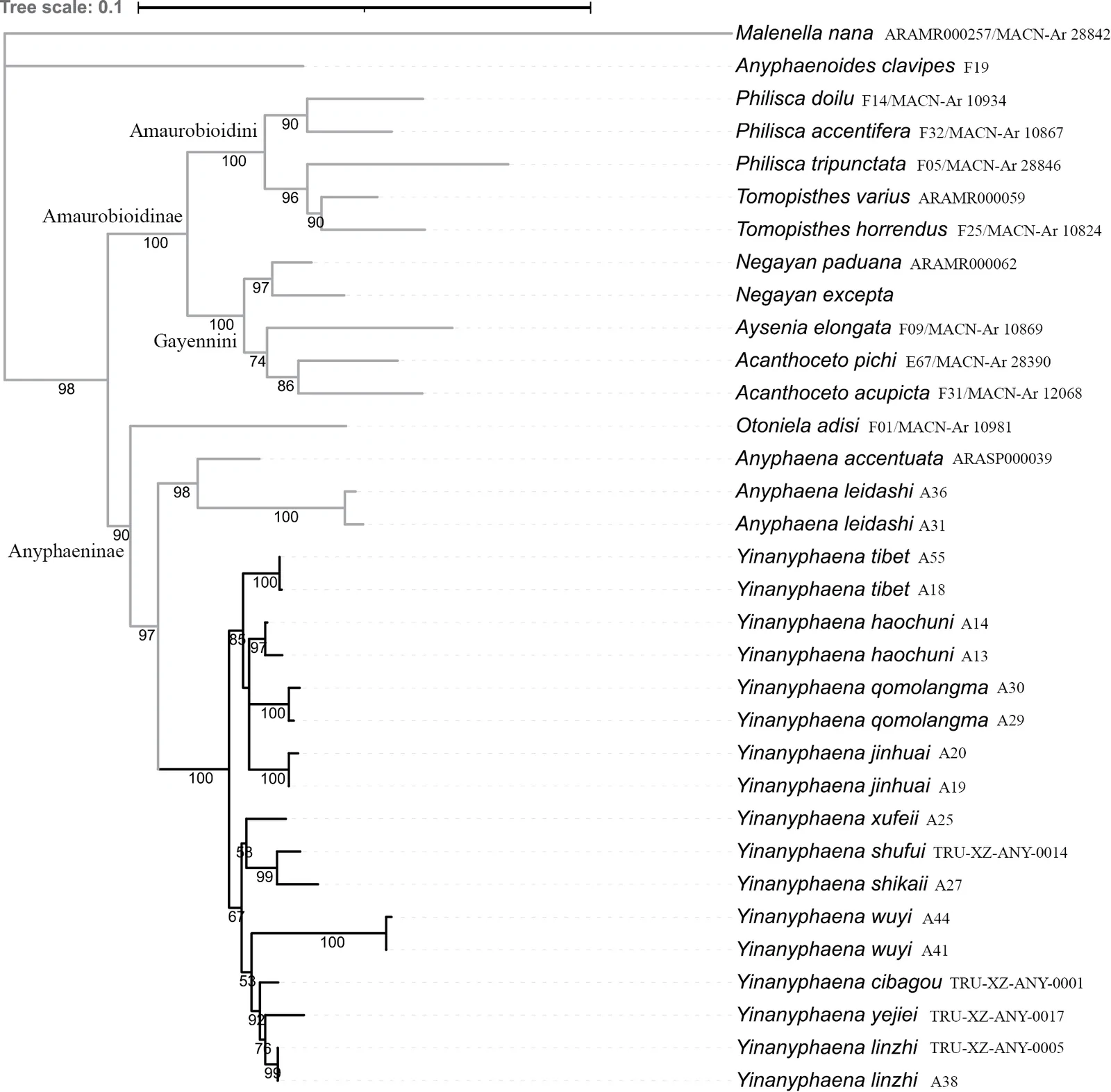
Maximum-likelihood phylogenetic tree inferred from concatenated sequences of four genes. Numbers at nodes indicate ML bootstrap support values. Bootstrap values ≥50 are shown.

The concatenated alignment of the four gene fragments resulted in a 2342 bp matrix. The lengths of individual gene matrices were as follows: COI (599 bp, 33 individuals), 28S (614 bp, 19 individuals), 18S (846 bp, 13 individuals), and H3 (283 bp, 24 individuals). The phylogenetic results are shown in Fig. 1. The subfamilies Anyphaeninae and Amaurobioidinae were recovered as reciprocally monophyletic, consistent with a previous study (Labarque et al. 2015). The new genus *Yinanyphaena* gen. nov., represented by 11 species (excluding *Y. rhynchophysa*), forms a group nested within Anyphaeninae as sister to *Anyphaena* and *Otoniela* Brescovit, 1997. This relationship is well supported by high bootstrap values (≥90), and the monophyly of the new genus is strongly supported by a bootstrap value of 100.

### Taxonomy

**Family Anyphaenidae Bertkau, 1878**

#### Genus Yinanyphaena

Taxon classificationAnimaliaAraneaeAnyphaenidae

Wang & Mi gen. nov.

BC8F002E-D875-5D84-B1C1-7C6C987ED42C

https://zoobank.org/49DB74FE-D889-46A2-B501-417BE40BD546

##### Type species.

*Anyphaena linzhi* Wang & Mi, 2024 from Xizang, China.

##### Etymology.

The generic name is a combination of Yin, referring to the late Prof. Changmin Yin, one of the pioneers of taxonomic study of Chinese spiders, and the genus name *Anyphaena* Sundevall, 1833; feminine in gender.

##### Diagnosis.

Males of *Yinanyphaena* gen. nov. resemble *Rathalos* Lin & Li, 2022 in the general shape of the bulb, especially the position and the form of the embolus, ventral tegular process and median apophysis, cymbium having two disto-prolateral macrosetae, but can be easily distinguished by the following: (1) cymbium lacks a dorsal apophysis (Figs 4C, 6C, 8C, 12C) vs has a dorsal apophysis (Lin et al. 2021: fig. 9A, C; Song and Zhu 1991: fig. 4); and (2) retrolateral tibial apophysis is bifurcated into two branches (Figs 4C, 6C, 8C, 12C) vs is just bifurcated at the distal end (Lin et al. 2021: fig. 9C; Song and Zhu 1991: fig. 4). Males of this genus are also similar to some members of the genus *Anyphaena* Sundevall, 1833, especially some of pectorosa-group species such as *A. epicardia*, *A. dulceae*, *A. rebecae* in the general shape of the palpal structure, but can be distinguished by the following: (1) median apophysis is inverted C-shaped (or 7-shaped) in retrolateral view and the ventral ramus of retrolateral tibial apophysis (vRTA) is tapered and bar-shaped in ventral view (Li et al. 2024: figs 2B, C, 3B, C; Lin et al. 2021: fig. 7B, C; Figs 4B, 4C, 6B, 6C, 8B, 8C, 12B, 12C) vs various in form (Rivera-Quiroz and Álvarez-Padilla 2023: figs 23A, D, 24A, D, 26A, D, 27A, D, 29A, D, 30A, D); and (2) cymbium has two disto-prolateral macrosetae (Li et al. 2024: figs 2B, 3B; Lin et al. 2021: fig. 7B; Figs 4B, 6B, 8B, 12B) vs lacking (Rivera-Quiroz and Álvarez-Padilla 2023: figs 23A, D, 24A, D, 26A, D, 27A, D, 29A, D, 30A, D). Females of *Yinanyphaena* gen. nov. are similar to *Sinophaena* Lin & Li, 2021 in the general shape of genitalia, but can be easily distinguished by the sclerotized atrium lacking wrinkles (Figs 4D, 6D, 8D, 10A, 10C, 12D) vs being membranous and having wrinkles (Lin et al. 2021: fig. 11A; Zhang and Song 2004: fig. 1B) and by the absence of a pair of slit-shaped lateral epigynal structures (Figs 4D, 6D, 8D, 10A, 10C, 12D) vs having such structure (Lin et al. 2021: fig. 11A; Zhang and Song 2004: fig. 1B).

##### Description.

**Figure 2.**
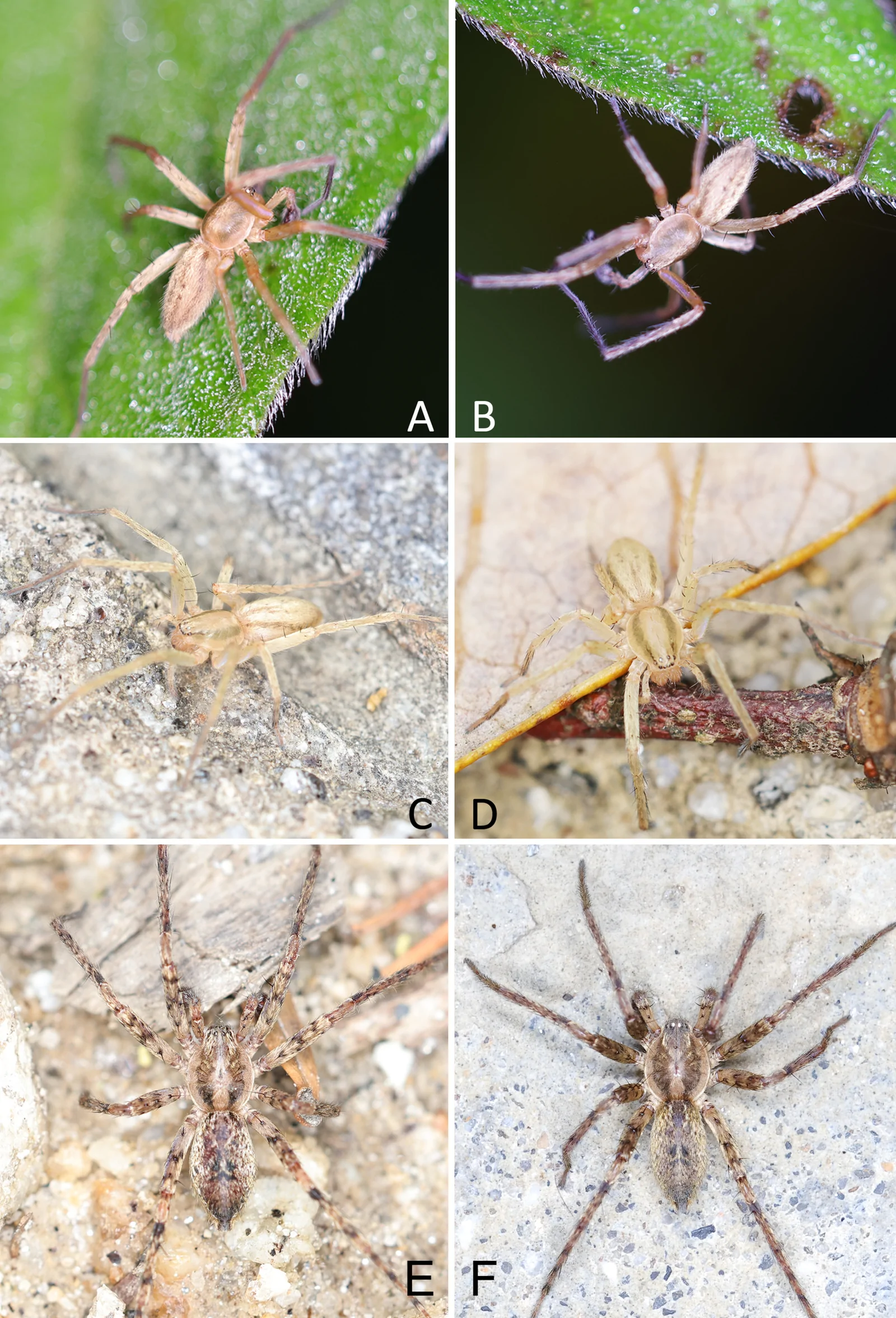
Living habitus of *Yinanyphaena jinhuai* sp. nov. (**E, F**) and *Y. qomolangma* sp. nov. (**A–D**). **A, B, E**. Male; **C, D, F**. Female: **A**. Dorso-lateral view; **B**. Lateral view; **C, D**. Dorso-anterior view; **E, F**. Dorsal view. Photographs by S.H. Luo.

Medium-sized spiders. Living habitus as shown in Fig. 2A–F. Carapace oval, with slightly elevated cephalon, and large symmetrical patch or pair of lateral longitudinal bands; fovea longitudinal. Chelicerae with four promarginal teeth and seven to nine retromarginal teeth. Endites slightly broadened distally and covered with dense dark setae on inner sides of anteromargins. Labium bearing dense dark setae on anteromargin. Sternum almost heart-shaped, mostly with symmetrical patches. Legs with ventral apophysis at base of coxae, and with red-brown patches in most species. Abdomen elongate-oval, dorsum mostly with two pairs of median irregular, dark patches; venter paler than dorsum.

***Palp***: patella with disto-prolateral apophysis; tibia about twice as long as wide, with disto-prolateral sub-triangular apophysis and baso-ventral lamellar apophysis; retrolateral tibial apophysis bifurcated to straight, bar-shaped ventral ramus, and dorsal ramus various in form; cymbium longer than wide, with two disto-prolateral macrosetae; bulb swollen; ventral tegular process prominent, antero-retrolaterally located; embolus flagelliform, partly visible; median apophysis enlarged at base, mostly inverted C-shaped or 7-shaped.

***Epigyne-vulva***: atrium located anteriorly, varying in form; copulatory openings beneath posterolateral portions of atrium; copulatory ducts varying in length, with accessory glands varying in form and position; spermathecae mostly oval or spherical, located posteriorly; fertilization ducts lamellar.

##### Composition.

The genus currently contains 12 species, including:

*Yinanyphaena cibagou* (Wang & Mi, 2024), comb. nov.;

*Y. haochuni* Wang & Mi, sp. nov.;

*Y. jinhuai* Wang & Mi, sp. nov.;

*Y. linzhi* (Wang & Mi, 2024), comb. nov.;

*Y. qomolangma* Wang & Mi, sp. nov.;

*Y. rhynchophysa* (Feng, Ma & Yang, 2012), comb. nov.;

*Y. shikaii* Wang & Mi, sp. nov.;

*Y. shufui* (Wang & Mi, 2024), comb. nov.;

*Y. tibet* (Lin & Li, 2021), comb. nov.;

*Y. wuyi* (Zhang, Zhu & Song, 2005), comb. nov.;

*Y. xufeii* Wang & Mi, sp. nov.;

*Y. yejiei* (Wang & Mi, 2024), comb. nov.

##### Distribution.

China (Fujian, Guizhou, Taiwan, Yunnan, Xizang) (Fig. 13).

##### Comments.

All new combinations are assigned to the new genus based on molecular evidence or strong morphological similarity. In the ML tree (Fig. 1), all except *Y. rhynchophysa*, for which molecular data are unavailable, are recovered within the *Yinanyphaena* clade. The three species *Y. cibagou*, *Y. shufui*, and *Y. yejiei*, though supported only by COI sequences, additionally exhibit a habitus and copulatory organ morphology that closely match those of the type species. This combined evidence justifies their formal transfer. For *Y. rhynchophysa*, despite the lack of phylogenetic placement, its close resemblance to *Y. wuyi* in both habitus and copulatory structures provides equally compelling morphological grounds for regarding it as congeneric.

#### Yinanyphaena haochuni

Taxon classificationAnimaliaAraneaeAnyphaenidae

Wang & Mi sp. nov.

759FF53A-92AC-5380-BD6D-955563BE444F

https://zoobank.org/742FA92B-5693-4053-8175-4E9712151400

Figs 3A–C, 4A–E

##### Type material.

***Holotype***: China • ♂ (TRU-ANY 0004); Xizang Autonomous Region, Rikaze City, Dingjie County, Chentang Town, Xiuxiongma Village, Jiuyanwenquan (27°55.01'N, 87°22.14'E, c. 2860 m), 26.V.2025, Cheng Wang et al. leg. ***Paratypes*** • 10♂12♀ (TRU-ANY 0005–0026); same data as for holotype; • 7♂4♀ (TRU-ANY 0027–0037); same locality as for holotype (27°55.09'N, 87°22.03'E, c. 2880 m), 21.V.2025, Cheng Wang et al. leg.

##### Etymology.

The specific name is a patronym in honour of Mr Haochun Chen, who helped on specimens collecting; noun (name) in the genitive case.

##### Diagnosis.

**Figure 3.**
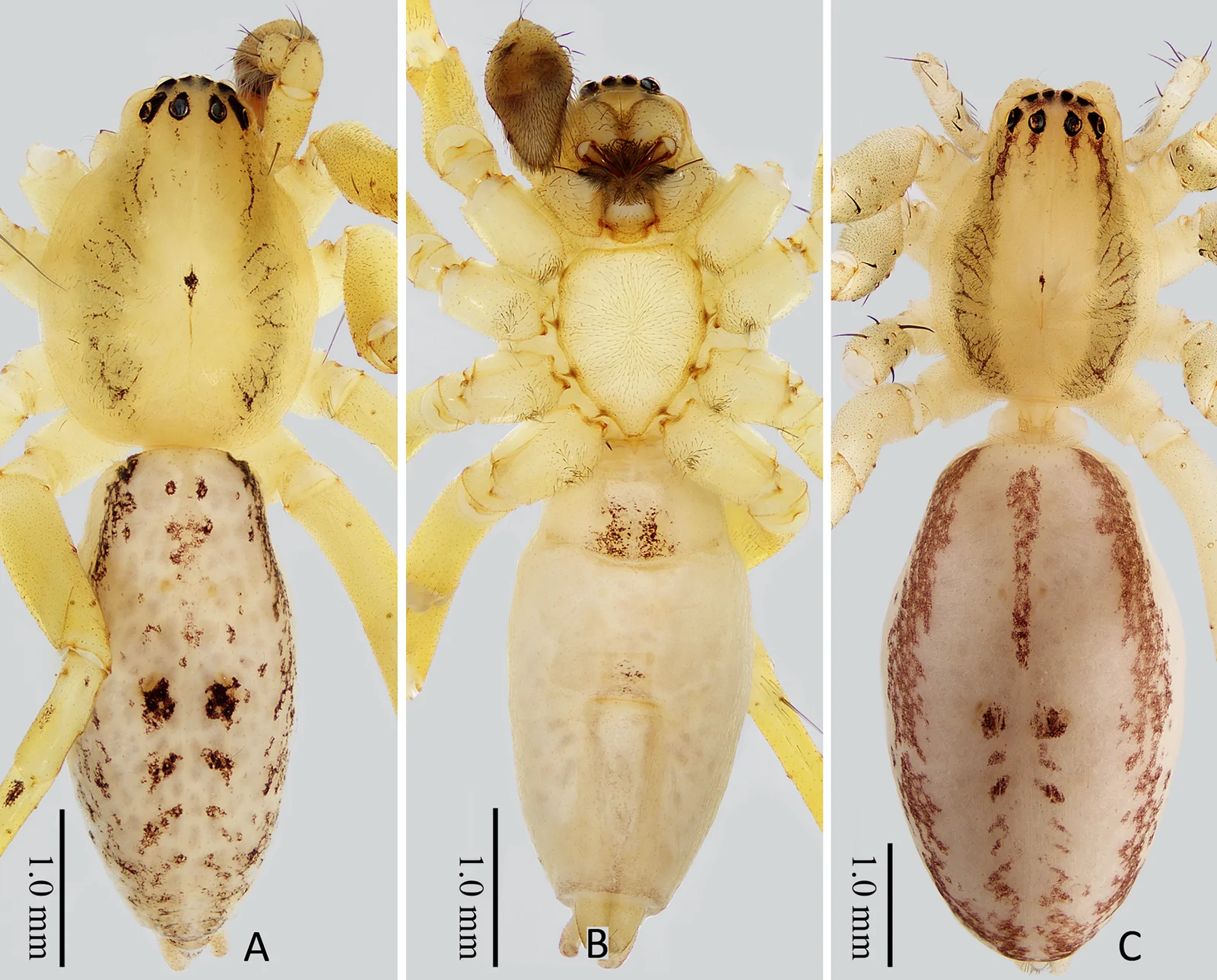
Habitus of *Yinanyphaena haochuni* Wang & Mi, sp. nov. **A, B**. Male holotype; **C**. Female paratype (TRU-ANY 0015); **A, C**. Dorsal view; **B**. Ventral view.

**Figure 4.**
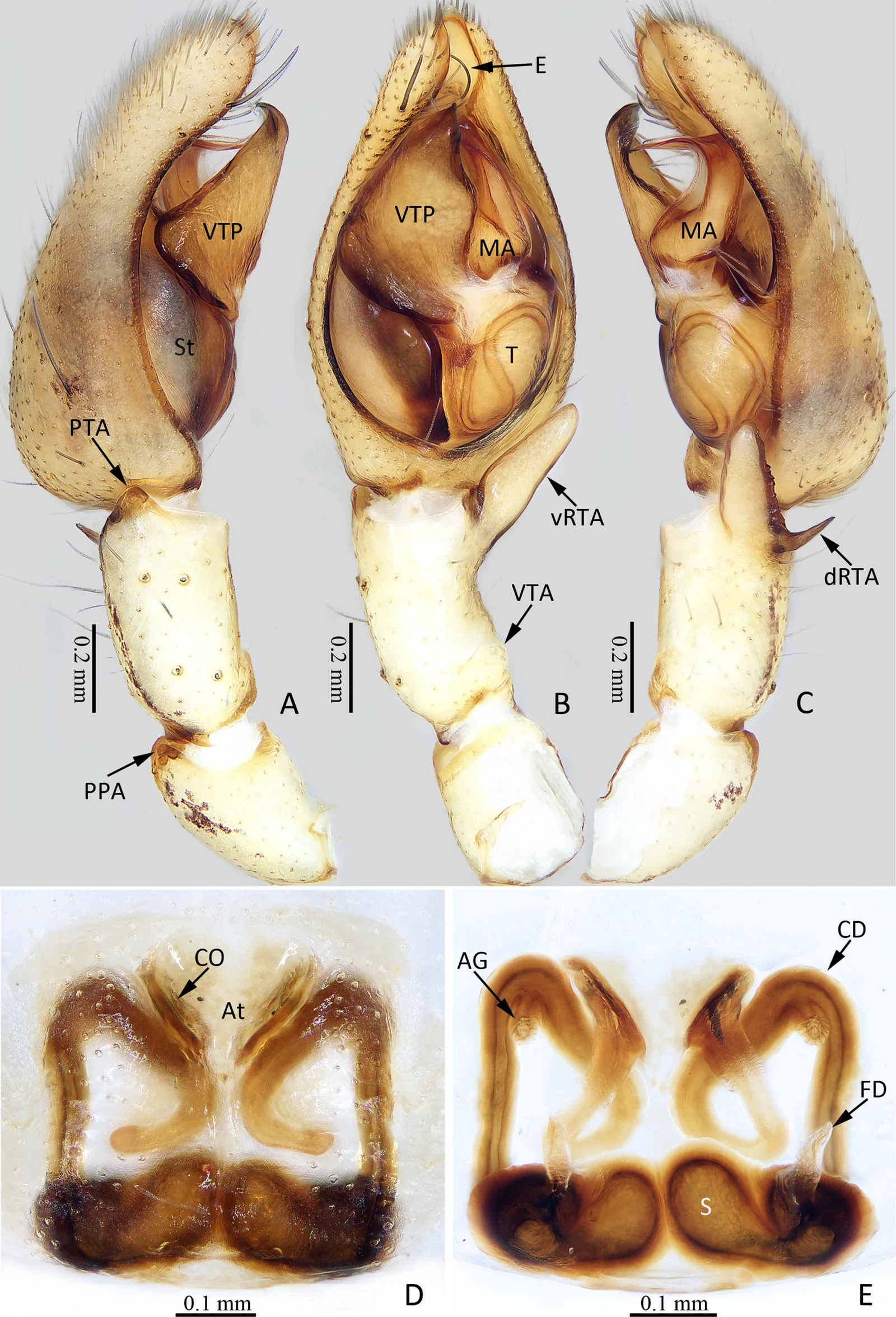
Copulatory organs of *Yinanyphaena haochuni* Wang & Mi, sp. nov., male holotype and female paratype (TRU-ANY 0015). **A**. Male palp, prolateral view; **B**. Ditto, ventral view; **C**. Ditto, retrolateral view; **D**. Epigyne, ventral view; **E**. Vulva, dorsal view.

*Yinanyphaena haochuni* sp. nov. can be easily distinguished from other congeners except *Y. qomolangma* sp. nov. by the paler habitus pattern and the presence of a pair of lateral patches extending through carapace, but can be distinguished from the latter by the following: (1) the dorsal ramus of the retrolateral tibial apophysis is straight and having a pointed tip (Fig. 4C), vs bent and with a blunt tip (Fig. 8C); (2) ventral tegular process is widest at base in ventral view (Fig. 4B), vs widest medially (Fig. 8B); (3) accessory glands are directed downward (Fig. 4E), vs upward (Fig. 8E); and (4) spermathecae are elongate-oval (Fig. 4E), vs nearly oval (Fig. 8E).

##### Description.

Male (holotype, Figs 3A, 3B, 4A–C). Total length 5.52. Carapace 2.33 long, 1.76 wide. Abdomen 3.20 long, 1.52 wide. Eye sizes: AME 0.08, ALE 0.13, PME 0.12, PLE 0.13, AME–AME 0.06, AME–ALE 0.03, PME–PME 0.14, PME–PLE 0.11, ALE–PLE 0.11. MOA long 0.27, anterior width 0.22, posterior width 0.36. Leg measurements: I 10.37 (2.65, 0.90, 2.96, 2.52, 1.34), II 8.27 (2.15, 0.82, 2.31, 1.91, 1.08), III 6.40 (1.80, 0.51, 1.76, 1.54, 0.79), IV 9.48 (2.54, 0.80, 2.34, 2.36, 1.44). Carapace oval, yellow, with slightly elevated cephalon, and pair of lateral, dark brown patches extending across whole surface; fovea longitudinal and yellow. Chelicerae yellow to brown, with four promarginal and nine retromarginal teeth. Endites yellow, broadened distally, bearing dense dark setae on inner portion of anterior margins. Labium yellow, bearing dark setae on anterior margin. Sternum about 1.3 times as long as wide. Legs yellow, spinous. Abdomen elongate, dorsum with dark brown patches and gray faint spots, and two pairs of median muscle depressions; venter pale to pale yellow.

***Palp*** (Fig. 4A–C): patella slightly longer than wide, with sub-triangular, disto-prolateral apophysis; tibia about twice as long as wide, with lamellar, near half-oval, baso-ventral apophysis; retrolateral tibial apophysis bifurcated, ventral ramus straight, tapered, blunted apically, more than half of tibial length, and with several tooth-like processes on dorsal margin, dorsal ramus spine-like, slightly curved at base; cymbium c. 1.7 times as long as wide; ventral tegular process widest at base, and curved dorsally at distal end; embolus flagelliform, partly visible; median apophysis inverted 7-shaped, enlarged at base.

**Female** (TRU-ANY 0015, Figs 3C, 4D, 4E). Total length 7.07. Carapace 2.59 long, 1.89 wide. Abdomen 4.22 long, 2.48 wide. Eye sizes: AME 0.10, ALE 0.15, PME 0.11, PLE 0.16, AME–AME 0.06, AME–ALE 0.03, PME–PME 0.18, PME–PLE 0.13, ALE–PLE 0.14. MOA long 0.31, anterior width 0.26, posterior width 0.41. Leg measurements: I 9.16 (2.35, 0.90, 2.62, 2.14, 1.15), II 8.27 (2.07, 0.92, 2.24, 1.94, 1.10), III 5.94 (1.82, 0.70, 1.36, 1.31, 0.75), IV 8.77 (2.55, 0.80, 2.12, 2.39, 0.91). Habitus (Fig. 3C) similar to that of male except dorsal abdomen with pair of longitudinal light red-brown bands and chelicerae with seven retromarginal teeth.

***Epigyne-vulva*** (Fig. 4D, E): epigynal plate almost as long as wide; atrium anteriorly located, almost heart-shaped, ridgy medially; copulatory openings beneath lateral portions of atrium; copulatory ducts slender, posterolaterally extending at origin, and then curving back and forming inverted V-shapes, with bar-shaped, median accessory glands directed downward; spermathecae elongate-oval, consisting of two sub-spherical portions; fertilization ducts extending almost anteriorly.

##### Distribution.

Known only from the type locality in Xizang, China (Fig. 13).

#### Yinanyphaena jinhuai

Taxon classificationAnimaliaAraneaeAnyphaenidae

Wang & Mi sp. nov.

B164B1EF-F191-5869-89A8-EC5C576B731B

https://zoobank.org/30834333-978A-4A02-9496-8FAFC4910023

**Figure 5.**
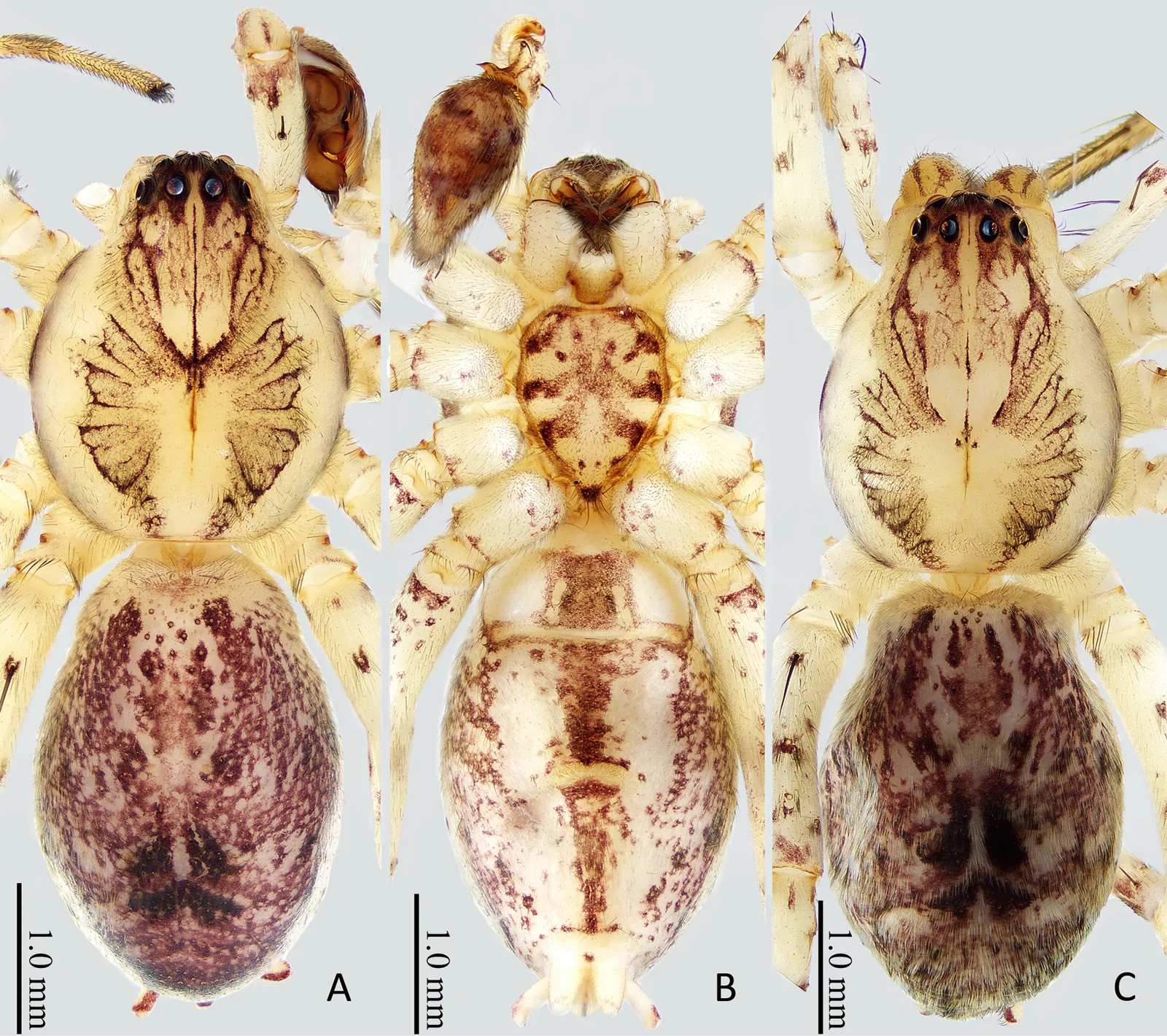
Habitus of *Yinanyphaena jinhuai* Wang & Mi, sp. nov. **A, B**. Male holotype; **C**. Female paratype (TRU-ANY 0040); **A, C**. Dorsal view; **B**. Ventral view.

**Figure 6.**
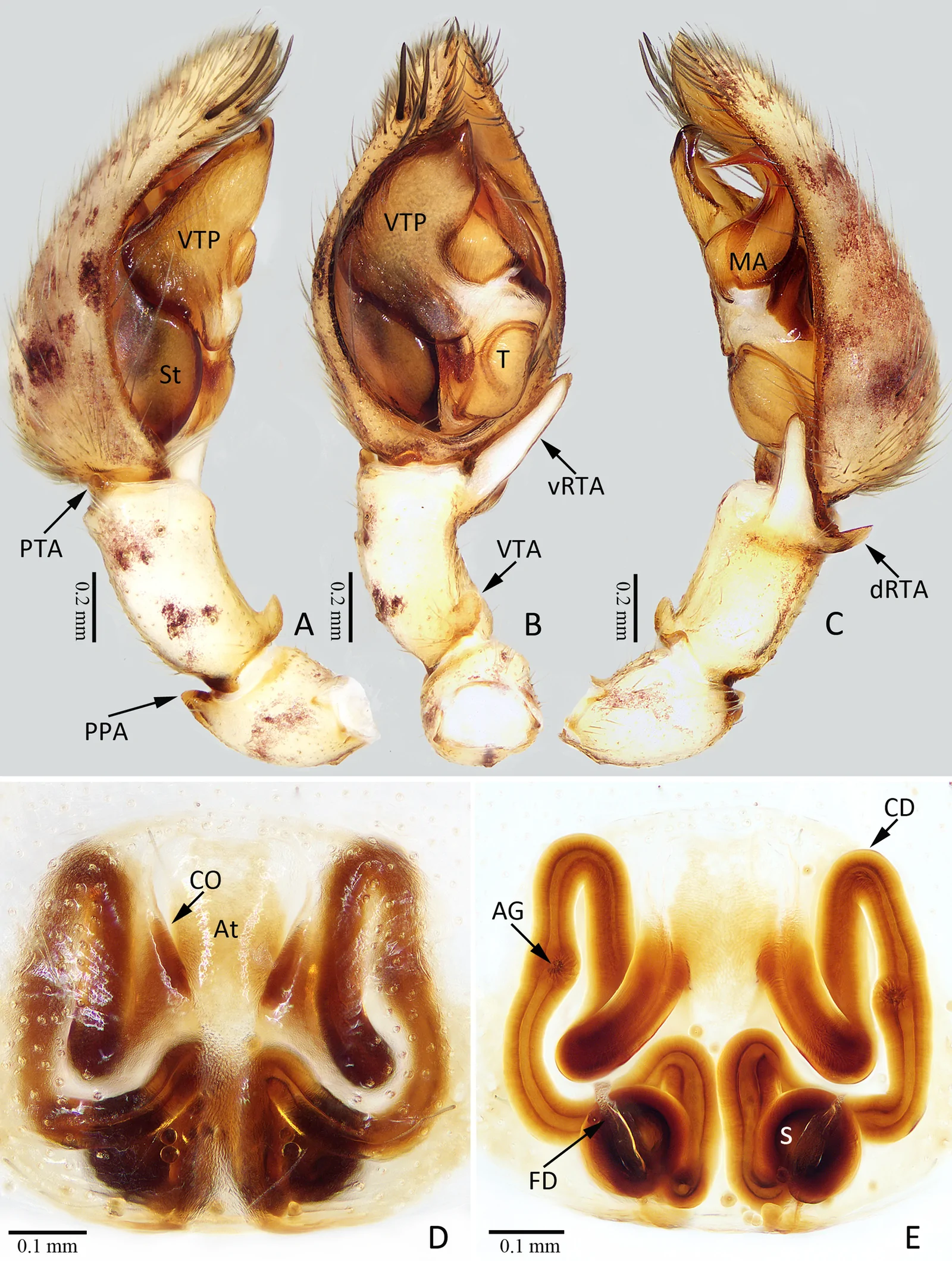
Copulatory organs of *Yinanyphaena jinhuai* Wang & Mi, sp. nov., male holotype and female paratype (TRU-ANY 0040). **A**. Male palp, prolateral view; **B**. Ditto, ventral view; **C**. Ditto, retrolateral view; **D**. Epigyne, ventral view; **E**. Vulva, dorsal view.

Figs 2E, 2F, 5A–C, 6A–E

##### Type material.

***Holotype***: China • ♂ (TRU-ANY 0038); Xizang Autonomous Region, Rikaze City, Jilong County, Jilong Town, Daman Village, Sima (28°25.78'N, 85°19.29'E, c. 3240 m), 24.V.2025, Xiaoqi Mi et al. leg. ***Paratypes*** • 1♂3♀ (TRU-ANY 0039–0042); same data as for holotype; • 1♂ (TRU-ANY 0043); Jilong Village (28°22.87'N, 85°19.60'E, c. 2790 m), 23.V.2025, Xiaoqi Mi et al. leg; • 1♀ (TRU-ANY 0044); Duofu Village (28°24.11'N, 85°17.92'E, c. 2900 m), 24.V.2025, Xiaoqi Mi et al. leg; • 1♀ (TRU-ANY 0045); Dingjie County, Chentang Town, Shalie Village (27°52.43'N, 87°24.87'E, c. 2390 m), 21.V.2025, Cheng Wang et al. leg; • 5♀ (TRU-ANY 0046–0050); Dingri County, Rongxia Town, Zuobude Village (28°01.72'N, 86°16.50'E, c. 2990 m), 24.V.2025, Cheng Wang et al. leg; • 1♀ (TRU-ANY 0051); Dingjie County, Chentang Town, Xiuxiongma Village (27°54.01'N, 87°23.23'E, c. 2600 m), 21.V.2025, Cheng Wang et al. leg; • 1♀ (TRU-ANY 0052); Jilong Village (28°23.48'N, 85°21.14'E, c. 2900 m), 26.V.2025, Xiaoqi Mi et al. leg.

##### Etymology.

The specific name is a patronym in honour of Mr Jinhua Li, one of the collectors of this species; noun (name) in the genitive case.

##### Diagnosis.

The species is very similar to *Y. tibet* (Lin & Li, 2021), comb. nov., in the habitus and copulatory organs, but can be easily distinguished by the following: (1) ventral tegular process is L-shaped in general in ventral view (Fig. 6B), vs near C-shaped (Lin et al. 2021: fig. 7B); (2) the dorsal ramus of the retrolateral tibial apophysis is straight, and less than one-third length of the ventral ramus (Fig. 6C), vs curved medially, and more than half length of the ventral ramus (Lin et al. 2021: fig. 7C); and (3) copulatory ducts extend beyond the antero-most edge of the atrium (Fig. 6E) vs not beyond (Lin et al. 2021: fig. 8B).

##### Description.

**Male** (holotype, Figs 5A, 5B, 6A–C). Total length 5.63. Carapace 2.56 long, 2.12 wide. Abdomen 2.95 long, 2.04 wide. Eye sizes: AME 0.08, ALE 0.14, PME 0.12, PLE 0.14, AME–AME 0.07, AME–ALE 0.04, PME–PME 0.14, PME–PLE 0.13, ALE–PLE 0.13. MOA long 0.27, anterior width 0.25, posterior width 0.37. Leg measurements: I 10.55 (2.70, 0.90, 3.12, 2.53, 1.30), II 9.33 (2.52, 0.96, 2.75, 2.05, 1.05), III 7.30 (2.14, 0.85, 2.03, 1.51, 0.77), IV 9.89 (2.60, 1.02, 2.62, 2.70, 0.95). Carapace oval, yellow in general, with slightly elevated cephalon, and large, symmetrical, irregular, red-brown marking in center; fovea longitudinal, reddish-brown. Chelicerae with four promarginal and nine retromarginal teeth. Endites pale yellow, longer than wide, bearing dense dark setae on inner portion of anterior margins. Labium pale yellow to red-brown, bearing anteromarginal dark setae. Sternum slightly longer than wide, with symmetrical red-brown markings. Legs slender, and spinous. Abdomen elongate-oval, dorsum red-brown, spotted, with two pairs of median muscle depressions followed by two pairs of irregular dark patches; venter pale to red-brown.

***Palp*** (Fig. 6A–C): patella slightly longer than wide, with short, sub-triangular disto-prolateral apophysis about one-fifth of patellar length; tibia about twice as long as wide, with lamellar, baso-ventral apophysis; retrolateral tibial apophysis bifurcated to straight, bar-shaped ventral ramus with blunt tip and lamellar dorsal ramus with pointed end; cymbium about 1.7 times as long as wide, with two disto-prolateral macrosetae; ventral tegular process generally L-shaped, distal end slightly curved inward; embolus flagelliform, partly visible; median apophysis inverted 7-shaped, enlarged at base.

**Female** (TRU-ANY 0040, Figs 5C, 6D, 6E). Total length 6.20. Carapace 2.83 long, 2.25 wide. Abdomen 3.27 long, 2.27 wide. Eye sizes: AME 0.10, ALE 0.16, PME 0.12, PLE 0.14, AME–AME 0.09, AME–ALE 0.06, PME–PME 0.19, PME–PLE 0.15, ALE–PLE 0.11. MOA long 0.33, anterior width 0.28, posterior width 0.43. Leg measurements: I 9.87 (2.63, 1.07, 2.79, 2.19, 1.19), II 9.30 (2.53, 1.05, 2.56, 2.01, 1.15), III 7.10 (2.18, 0.84, 1.62, 1.74, 0.72), IV 10.26 (2.84, 0.97, 2.47, 2.51, 1.47). Habitus (Fig. 5C) similar to that of male except chelicerae with seven retromarginal teeth.

***Epigyne-vulva*** (Fig. 6D, E): epigynal plate about as long as wide, atrium about inverted trapeziform, slightly ridgy; copulatory openings beneath lateral portions of atrium; copulatory ducts slender, forming complex paths, with antero-median, indistinct accessory glands; spermathecae spherical, apart from each other about 2/3 their diameter; fertilization ducts diverging.

##### Distribution.

Known only from the type localities in Xizang, China (Fig. 13).

#### Yinanyphaena qomolangma

Taxon classificationAnimaliaAraneaeAnyphaenidae

Wang & Mi sp. nov.

8F365861-EA21-5784-A813-A19AA38FDBFE

https://zoobank.org/094CE9EE-D0AB-403E-B15B-0C2721E36C39

**Figure 7.**
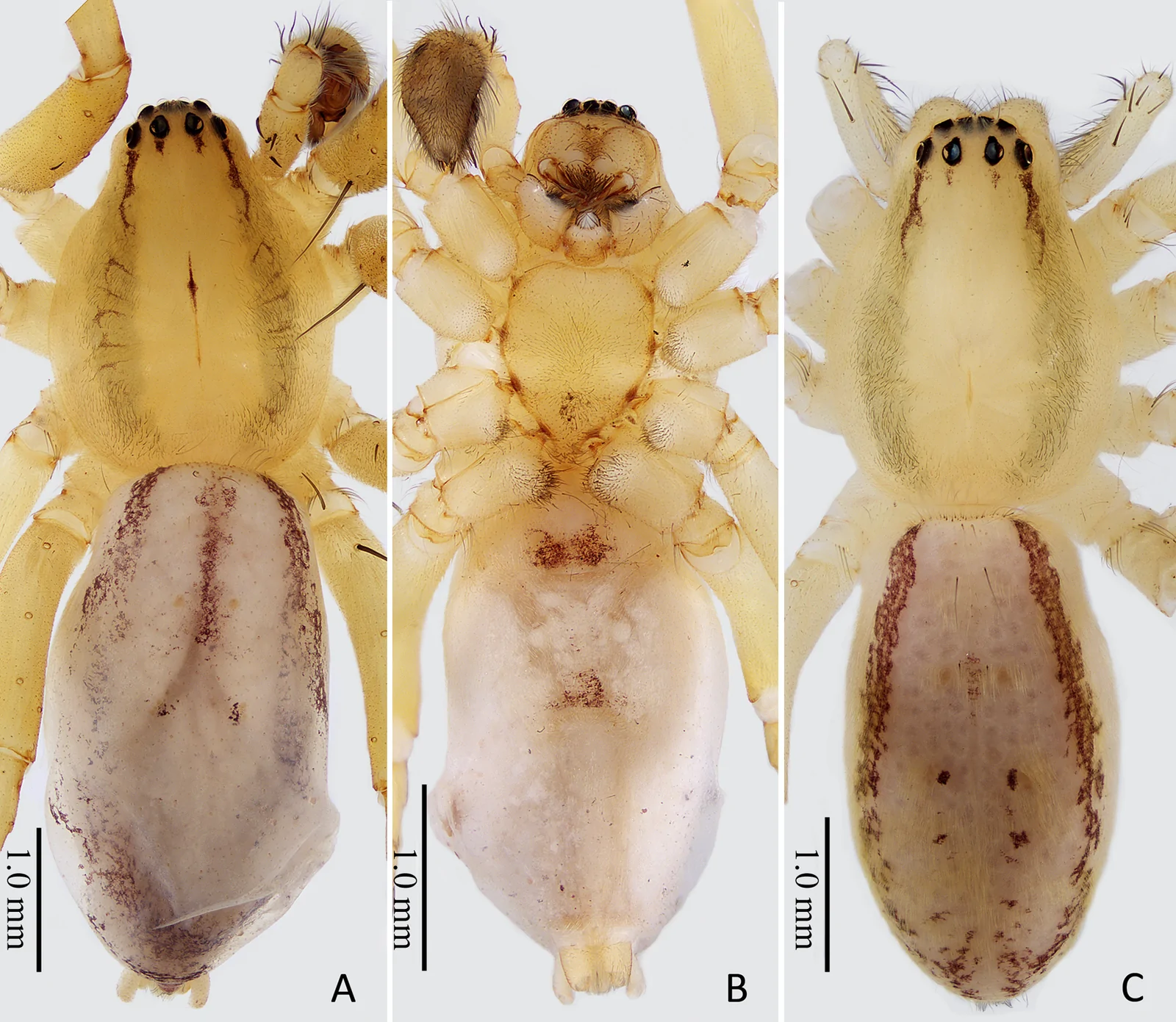
Habitus of *Yinanyphaena qomolangma* Wang & Mi, sp. nov. **A, B**. Male holotype; **C**. Female paratype (TRU-ANY 0055); **A, C**. Dorsal view; **B**. Ventral view.

**Figure 8.**
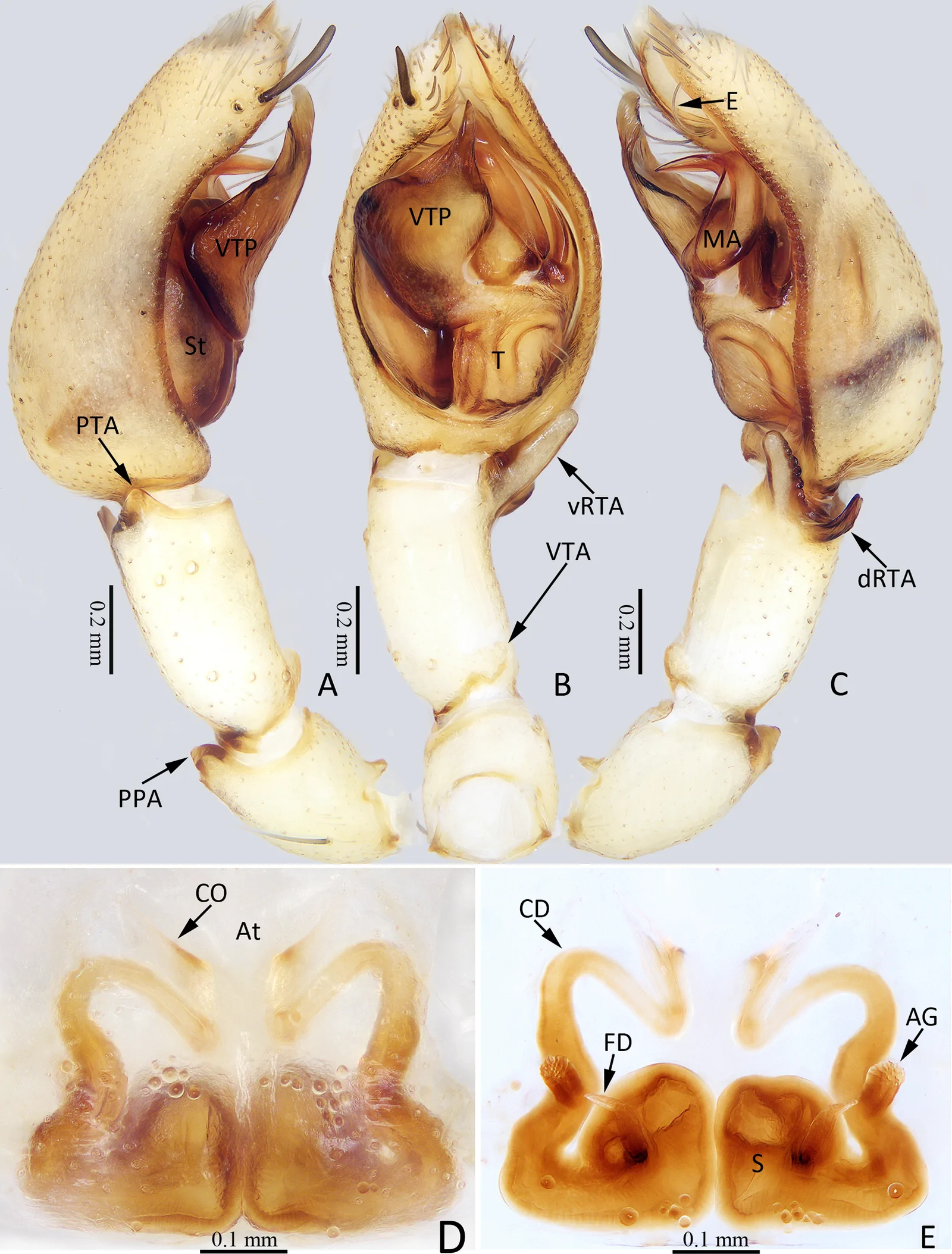
Copulatory organs of *Yinanyphaena qomolangma* Wang & Mi, sp. nov., male holotype and female paratype (TRU-ANY 0055). **A**. Male palp, prolateral view; **B**. Ditto, ventral view; **C**. Ditto, retrolateral view; **D**. Epigyne, ventral view; **E**. Vulva, dorsal view.

Figs 2A–D, 7A–C, 8A–E

##### Type material.

***Holotype***: China • ♂ (TRU-ANY 0053); Xizang Autonomous Region, Nielamu County, Zhangmu Town, Lixin Village (27°56.92'N, 85°58.40'E, c. 2510 m), 22.V.2025, Xiaoqi Mi et al. leg. ***Paratypes*** • 1♂1♀ (TRU-ANY 0054–0055); same data as for holotype; • 3♂2♀ (TRU-ANY 0056–0060); Dingri County, Rongxia Town, Zuobude Village (28°01.72'N, 86°16.50'E, c. 2990 m), 24.V.2025, Cheng Wang et al. leg; • 1♂ (TRU-ANY 0061); Nielamu County, Zhangmu Town, Zhangmu Village (27°59.53'N, 85°59.71'E, c. 2320 m), night of 21.V.2025, Xiaoqi Mi et al. leg.

##### Etymology.

The specific name is after the type locality, Qomolangma National Nature Reserve; noun in apposition.

##### Diagnosis.

The species is very similar to *Y. haochuni* sp. nov. in having a very similar habitus pattern, but can be easily distinguished by the following: (1) the dorsal ramus of the retrolateral tibial apophysis is curved medially and having a blunt tip (Fig. 8C), vs straight and having a pointed tip (Fig. 4C); (2) ventral tegular process is widest medially in ventral view (Fig. 8B), vs widest at the base (Fig. 4B); (3) accessory glands are directed upward (Fig. 8E), vs downward (Fig. 4E); and (4) spermathecae are nearly oval (Fig. 8E) vs elongate-oval (Fig. 4E).

##### Description.

**Male** (holotype, Figs 7A, 7B, 8A–C). Total length 6.16. Carapace 2.66 long, 2.00 wide. Abdomen 3.64 long, 2.06 wide. Eye sizes: AME 0.10, ALE 0.12, PME 0.10, PLE 0.12, AME–AME 0.06, AME–ALE 0.02, PME–PME 0.15, PME–PLE 0.08, ALE–PLE 0.13. MOA long 0.21, anterior width 0.23, posterior width 0.36. Leg measurements: I 11.54 (2.85, 1.05, 3.25, 2.90, 1.49), II 10.27 (2.68, 1.09, 2.84, 2.40, 1.26), III 6.75 (2.07, 0.73, 1.72, 1.45, 0.78), IV 10.05 (2.75, 0.84, 2.75, 2.69, 1.02). Carapace yellow, near oval, cephalon slightly elevated, with two pairs of longitudinal, red-brown stripes behind eye field and pair of longitudinal, indistinct, brown lateral patches extending through whole surface; fovea longitudinal, anteriorly fused with longitudinal stripe. Chelicerae yellow, with four promarginal and eight retromarginal teeth. Endites pale to yellow, expanding laterally at distal portions, covered with dense, dark setae on inner sides of antero-margins. Labium colored as endites. Sternum yellow, about 1.2 times as long as wide, covered with dense, thin setae posteromedially. Legs pale to yellow, covered with dense ventral setae at base of coxae. Abdomen elongate, dorsum with pair of lateral red-brown patches extending across whole surface and central, longitudinal stripe extending about anterior 1/3, as well as two pairs of median muscle depressions; ventral pale in general.

***Palp*** (Fig. 8A–C): patella slightly longer than wide, with short, disto-prolateral apophysis with blunt tip; tibia about twice as long as wide, with lamellar, baso-ventral apophysis and subtriangular disto-prolateral apophysis; retrolateral tibial apophysis bifurcated to straight ventral ramus bearing several sclerotized processes on dorsal margin, and dorsal ramus slightly curved medially and almost equal in width; cymbium about 1.63 times as long as wide, with two distoprolateral macrosetae; ventral tegular process widest medially, with anterior, narrow extension; embolus flagelliform partly visible; median apophysis enlarged at base, almost inverted 7-shaped.

**Female** (TRU-ANY 0055, Figs 7C, 8D, 8E). Total length 5.71. Carapace 2.51 long, 1.91 wide. Abdomen 3.13 long, 1.79 wide. Eye sizes: AME 0.11, ALE 0.14, PME 0.11, PLE 0.15, AME–AME 0.04, AME–ALE 0.03, PME–PME 0.17, PME–PLE 0.13, ALE–PLE 0.11. MOA long 0.28, anterior width 0.25, posterior width 0.39. Leg measurements: I 9.93 (2.70, 0.99, 2.74, 2.35, 1.15), II 8.34 (2.25, 0.85, 2.34, 1.95, 0.95), III 6.18 (1.81, 0.83, 1.54, 1.35, 0.65), IV 9.87 (3.18, 0.88, 2.33, 2.59, 0.89).

Habitus (Fig. 7C) similar to that of male except chelicerae with seven retromarginal teeth.

***Epigyne-vulva*** (Fig. 8D, E): epigynal plate wider than long; atrium anteriorly located, without distinct anterior border; copulatory openings beneath posterolateral portions of atrium; copulatory ducts posteriorly extending, then curved to almost two continuous C-shaped curves, with antero-obliquely extending, bar-shaped accessory glands medially; spermathecae almost oval, touching each other; fertilization ducts diverging.

##### Distribution.

Known only from the type localities in Xizang, China (Fig. 13).

#### Yinanyphaena shikaii

Taxon classificationAnimaliaAraneaeAnyphaenidae

Wang & Mi sp. nov.

0388F5EB-6A15-5626-A84C-467B0E2DA659

https://zoobank.org/8E09797D-9071-437E-B270-8A2E1E213075

**Figure 9.**
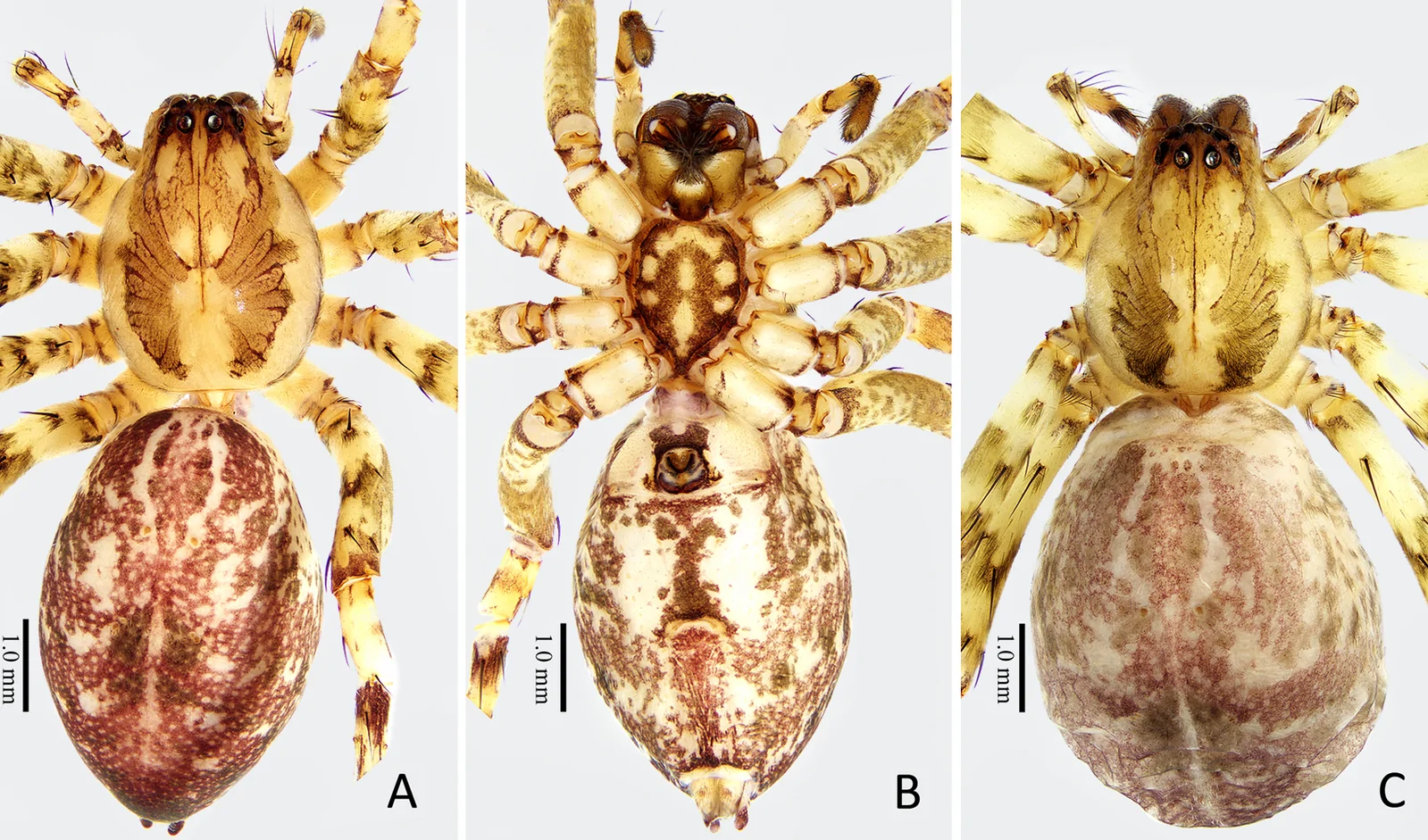
Female of *Yinanyphaena* spp., holotypes. **A, B**. *Y. shikaii* Wang & Mi, sp. nov.; **C**. *Y. xufeii* Wang & Mi, sp. nov.; **A, C**. Habitus, dorsal view; **B**. Ditto, ventral view.

**Figure 10.**
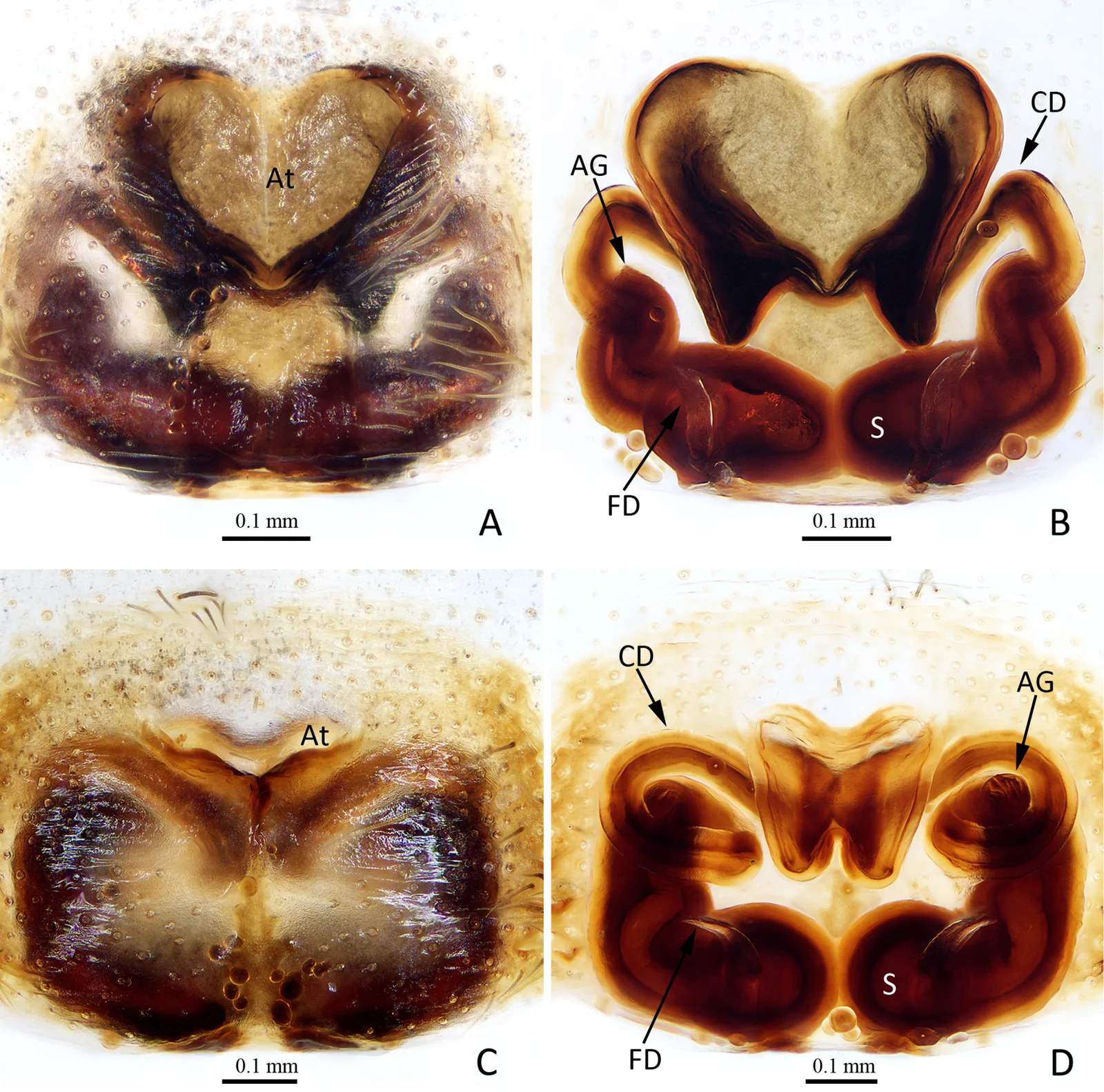
Epigyne-vulva of *Yinanyphaena* spp., holotypes. **A, B**. *Y. shikaii* Wang & Mi, sp. nov.; **C, D**. *Y. xufeii* Wang & Mi, sp. nov.; **A, C**. Epigyne, ventral view; **B, D**. Vulva, dorsal view.

Figs 9A, 9B, 10A, 10B

##### Type material.

***Holotype***: China • ♀ (TRU-ANY 0062); Xizang Autonomous Region, Rikaze City, Dingjie County, Chentang Town, Natang Village (27°50.47'N, 87°26.28'E, c. 2390 m), 27.V.2025, Cheng Wang et al. leg. ***Paratypes*** • 2♀ (TRU-ANY 0063–0064); same data as for holotype.

##### Etymology.

The specific name is a patronym in honour of Mr Shikai Li, one of the collectors of our spider surveys from Qomolangma National Nature Reserve in 2024, 2025; noun (name) in genitive case.

##### Diagnosis.

*Yinanyphaena shikaii* sp. nov. closely resembles *Y. rhynchophysa* (Feng, Ma & Yang, 2012), comb. nov. in having similar genitalia, but can be distinguished by the atrium occupying about half of the epigynal length and width (Fig. 10A) vs about 1/3 of the epigynal length and 1/4 of the epigynal width (Feng et al. 2012: fig. 8) and by the oval spermathecae (Fig. 10B) vs spherical (Feng et al. 2012: fig. 9).

##### Description.

**Female** (holotype, Figs 9A, 9B, 10A, 10B). Total length 7.79. Carapace 3.13 long, 2.38 wide. Abdomen 4.48 long, 3.04 wide. Eye sizes: AME 0.11, ALE 0.20, PME 0.14, PLE 0.20, AME–AME 0.08, AME–ALE 0.02, PME–PME 0.18, PME–PLE 0.16, ALE–PLE 0.09. MOA long 0.33, anterior width 0.31, posterior width 0.47. Leg measurements: I 12.25 (3.24, 1.25, 3.42, 2.89, 1.45), II 11.37 (3.09, 1.25, 3.10, 2.63, 1.30), III 8.89 (2.51, 1.03, 2.24, 2.15, 0.96), IV 11.93 (3.39, 1.15, 2.88, 3.16, 1.35). Carapace yellow, with slightly elevated cephalon, and large, symmetrical red-brown patch extending from behind eye field to posterior end; fovea longitudinal. Chelicerae dark-red, with four promarginal and seven retromarginal teeth. Endites deep yellow, longer than wide, bearing dense dark setae on inner portion of anterior margins. Labium darker than endites. Sternum red-brown, about 1.5 times as long as wide, with several yellow patches. Legs yellow in general, spinous, with dark brown or red-brown patches. Abdomen elongate-oval, dorsum red-brown, spotted, with pair of dark brown patches medially and two pairs of anteromedian muscle depressions; venter paler than dorsum.

***Epigyne-vulva*** (Fig. 10A, B): epigynal plate slightly wider than long, atrium anteriorly located, heart-shaped, about half of epigynal width; copulatory openings beneath posterolateral portions of atrium; copulatory ducts twisted, with stout median accessory glands directed upward; spermathecae oval, transversely extending, touching each other; fertilization ducts lamellar.

**Male**. Unknown.

##### Distribution.

Known only from the type locality in Xizang, China (Fig. 13).

#### Yinanyphaena tibet

Taxon classificationAnimaliaAraneaeAnyphaenidae

(Lin & Li, 2021) comb. nov.

0D005725-0831-5CD8-A996-46DC0E9257C8

Anyphaena tibet Lin & Li, in Lin et al. 2021: 98, figs 7A–C, 8A, B, 13B, 14G, H (D♂♀).

##### Material examined.

China • 4♂8♀ (TRU-ANY 0065–0076); Xizang Autonomous Region, Rikaze City, Dingjie County, Chentang Town, Xiuxiongma Village, Jiuyanwenquan (27°55.09'N, 87°22.03'E, c. 2880 m), 16.V.2025, Cheng Wang et al. leg. • 3♂5♀ (TRU-ANY 0077–0084); same locality as above (27°55.01'N, 87°22.14'E, c. 2860 m), 26.V.2025, Cheng Wang et al. leg.; • 1♀ (TRU-ANY 0085); Chentang Town, Natang Village (27°50.47'N, 87°26.28'E, c. 2390 m), 27.V.2025, Cheng Wang et al. leg.

##### Diagnosis and description.

See Lin et al. (2021).

##### Distribution.

China (Xizang) (Fig. 13).

#### Yinanyphaena wuyi

Taxon classificationAnimaliaAraneaeAnyphaenidae

(Zhang, Zhu & Song, 2005) comb. nov.

94302376-4F84-5E9B-8C4B-C7DFF670ABA5

Figs 11A–C, 12A–E

Anyphaena wuyi Zhang, Zhu & Song, 2005: 2, figs 1–10 (D♂♀); Chen, 2010: 70, figs 1–6 (♂♀); Chen, 2012: 36, figs 10A–E, pl. 3C, D (♂♀); Zhang & Wang, 2017: 63, fig. 5 (♂♀).

##### Material examined.

China • 1♂1♀ (TRU-ANY 0086–0087); Guizhou, Yinjiang Tujia and Miao Autonomous County, Ziwei Township, Fanjing Mountain National Nature Reserve, Huguosi (27°54.54'N, 108°46.57'E, c. 1660 m), 25.XI.2025; Cheng Wang et al. leg.

**Figure 11.**
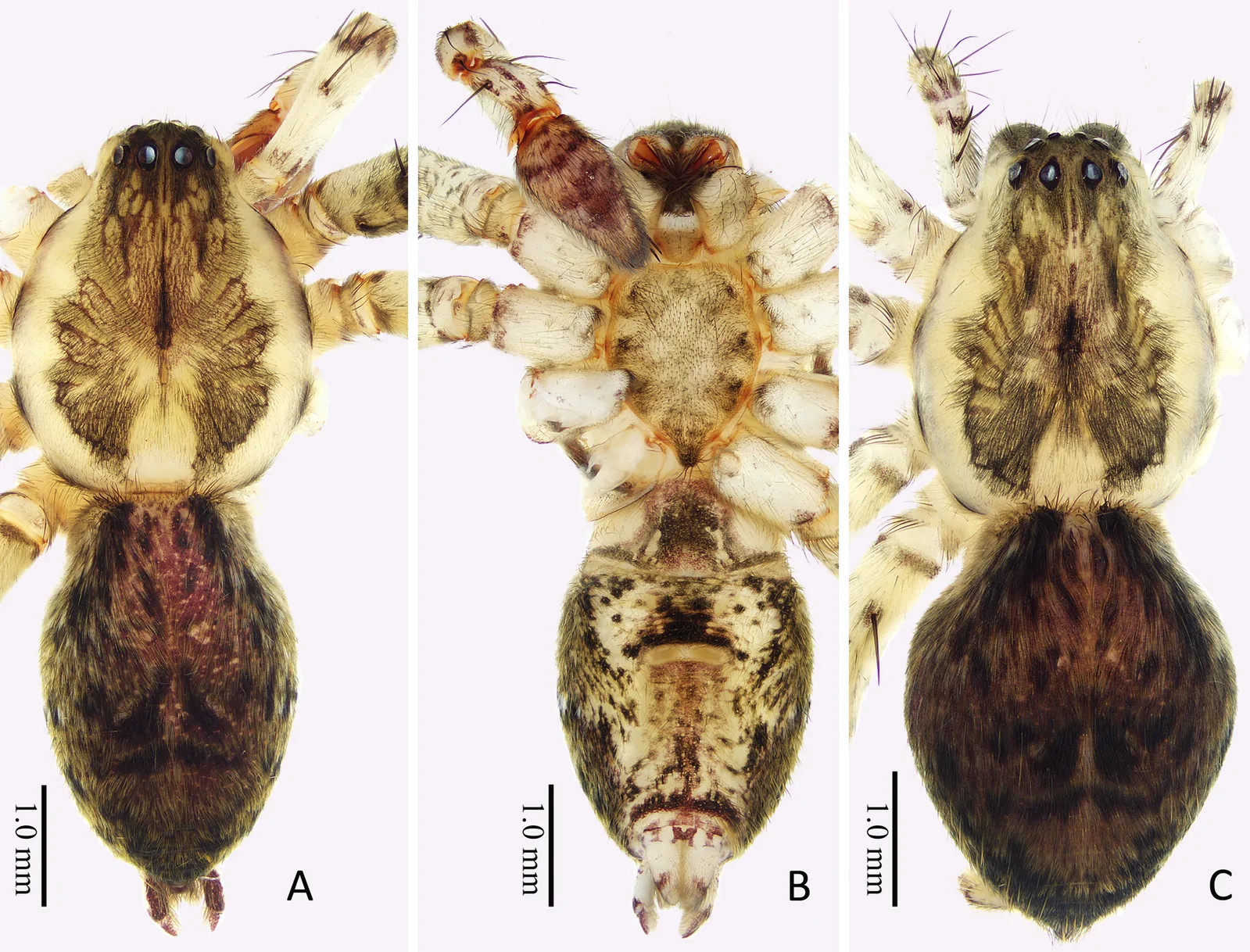
Habitus of *Yinanyphaena wuyi* (Zhang, Zhu & Song, 2005), comb. nov. **A, B**. Male (TRU-ANY 0086); **C**. Female (TRU-ANY 0087); **A, C**. Dorsal view; **B**. Ventral view.

**Figure 12.**
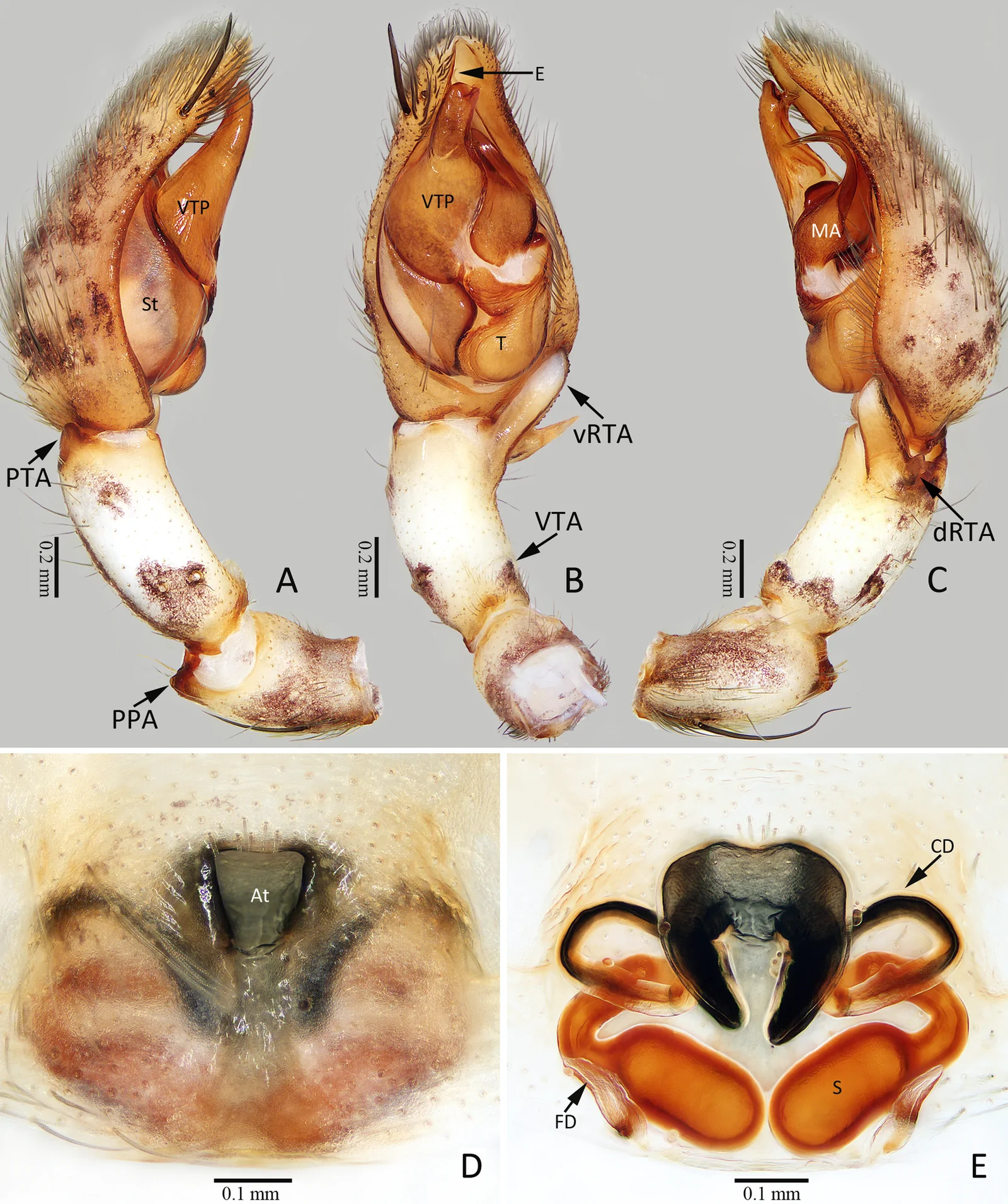
Copulatory organs of *Yinanyphaena wuyi* (Zhang, Zhu & Song, 2005), comb. nov., male (TRU-ANY 0086) and female (TRU-ANY 0087). **A**. Male palp, prolateral view; **B**. Ditto, ventral view; **C**. Ditto, retrolateral view; **D**. Epigyne, ventral view; **E**. Ditto, dorsal view.

##### Diagnosis and description.

See Zhang et al. (2005).

##### Distribution.

**Figure 13.**
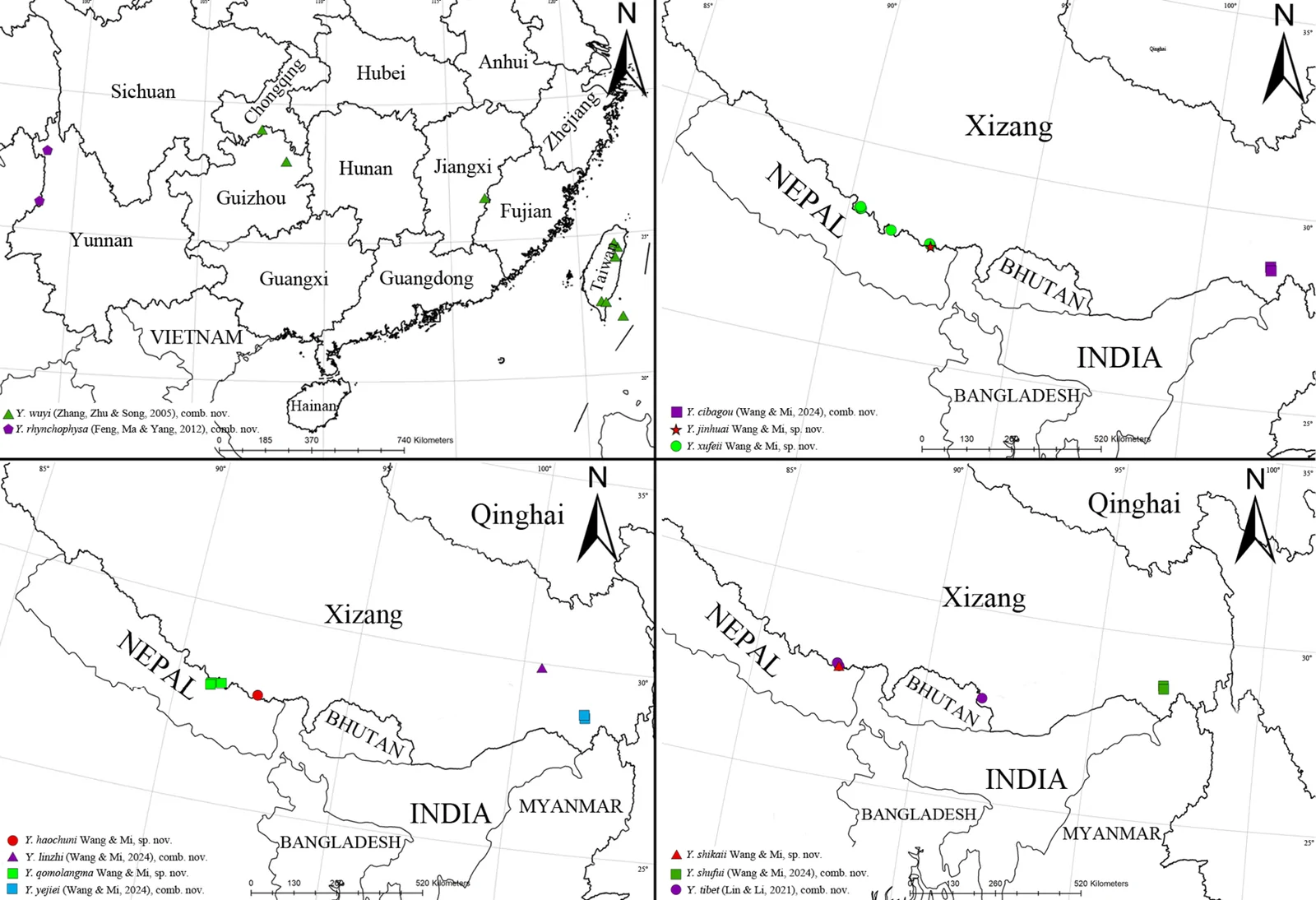
The distributional records of *Yinanyphaena* species.

China (Fujian, Guizhou, Taiwan) (Fig. 13).

#### Yinanyphaena xufeii

Taxon classificationAnimaliaAraneaeAnyphaenidae

Wang & Mi sp. nov.

A78BE9E3-1159-50A2-BD03-FC710520C3B1

https://zoobank.org/E8CAB91A-1E6F-48BB-862C-FD56BE66F870

Figs 9C, 10C, 10D

##### Type material.

***Holotype***: China • ♀ (TRU-ANY 0088); Xizang Autonomous Region, Rikaze City, Dingjie County, Chentang Town, Natang Village (27°50.47'N, 87°26.28'E, c. 2390 m), 27.V.2025, Cheng Wang et al. leg. ***Paratype*** • 1♀ (TRU-ANY 0089); same data as for holotype.

##### Etymology.

The specific name is a patronym in honour of Mr Xufei Zhu, one of the collectors of this species; noun (name) in the genitive case.

##### Diagnosis.

*Yinanyphaena xufeii* sp. nov. is closely similar to *Y. shufui* (Wang & Mi, 2024), comb. nov., in having a reduced atrium, but can be easily distinguished by the following: (1) atrium is less than half of the epigynal width (Fig. 10C) vs more than half (Li et al. 2024: fig. 5E); and (2) copulatory ducts are coiled into a circle at the median portions (Fig. 10D) vs not coiled (Li et al. 2024: fig. 5F).

##### Description.

**Female** (holotype, Figs 9C, 10C, 10D). Total length 7.94. Carapace 3.04 long, 2.55 wide. Abdomen 4.88 long, 3.92 wide. Eye sizes: AME 0.13, ALE 0.21, PME 0.17, PLE 0.19, AME–AME 0.08, AME–ALE 0.03, PME–PME 0.19, PME–PLE 0.17, ALE–PLE 0.14. MOA long 0.50, anterior width 0.34, posterior width 0.52. Leg measurements: I 12.52 (3.30, 1.25, 3.46, 2.90, 1.61), II 11.40 (3.08, 1.15, 3.11, 2.68, 1.38), III 9.00 (2.55, 1.14, 2.19, 2.15, 0.97), IV 12.22 (3.39, 1.30, 2.99, 3.27, 1.27). Carapace yellow, with slightly elevated cephalon and symmetrical brown patch extending across whole surface; fovea longitudinal. Chelicerae red-brown, with four promarginal and eight retromarginal teeth. Endites longer than wide, bearing dense dark setae on inner portion of anterior margins. Labium darker than endites. Sternum oval-shaped, with irregular brown patches. Legs yellow, spinous, with dark brown or red-brown patches. Abdomen oval, dorsum mingled with irregular pale and red-brown patches, with two pairs of median muscle depressions; venter pale red-brown.

***Epigyne-vulva*** (Fig. 10C, D): epigynal plate wider than long, atrium anteriorly located, slit-shaped, about 1/3 of epigynal width; copulatory openings beneath lateral sides of atrium; copulatory ducts broadened at base, and then gradually thickened, coiled into circle and with short, anteriorly extending accessory glands on median portions; spermathecae spherical, touching each other; fertilization ducts diverging.

**Male**. Unknown.

##### Distribution.

Known only from the type locality in Xizang, China (Fig. 13).

## Supplementary Material

XML Treatment for Yinanyphaena

XML Treatment for Yinanyphaena haochuni

XML Treatment for Yinanyphaena jinhuai

XML Treatment for Yinanyphaena qomolangma

XML Treatment for Yinanyphaena shikaii

XML Treatment for Yinanyphaena tibet

XML Treatment for Yinanyphaena wuyi

XML Treatment for Yinanyphaena xufeii

## Additional information

### Artificial Intelligence (AI) use

The authors accept full responsibility for the content of the manuscript, including the disclosure of any use of AI.

No AI tools were used in the preparation of this manuscript.

### Author ORCIDs

J. Zhang https://orcid.org/0009-0002-9726-4485

Z. Feng https://orcid.org/0000-0002-5809-5984

J. Gan https://orcid.org/0000-0001-6899-3705

C. Wang https://orcid.org/0000-0003-1831-0579

X. Mi https://orcid.org/0000-0003-1744-3855

### Data availability

All of the data that support the findings of this study are available in the main text.
